# Household economy, forest dependency & opportunity costs of conservation in eastern rainforests of Madagascar

**DOI:** 10.1038/sdata.2018.225

**Published:** 2018-10-23

**Authors:** Mahesh Poudyal, O. Sarobidy Rakotonarivo, Julie H. Razafimanahaka, Neal Hockley, Julia P. G. Jones

**Affiliations:** 1 School of Environment, Natural Resources and Geography, Bangor University, Bangor, UK; 2 Ecosystem Services for Poverty Alleviation (ESPA) Programme Directorate, Edinburgh, UK; 3 Biological and Environmental Sciences, Faculty of Natural Sciences, University of Stirling, Stirling, UK; 4 Madagasikara Voakajy, Antananarivo, Madagascar

**Keywords:** Socioeconomic scenarios, Sustainability

## Abstract

The Government of Madagascar is trying to reduce deforestation and conserve biodiversity through creating new protected areas in the eastern rainforests. While this has many benefits, forest use restriction may bring costs to farmers at the forest frontier. We explored this through a series of surveys in five sites around the Corridor Ankeniheny Zahamena new protected area and adjacent national parks. In phase one a stratified random sample of 603 households completed a household survey covering demographic and socio-economic characteristics, and a choice experiment to estimate the opportunity costs of conservation. A stratified sub-sample (n = 171) then completed a detailed agricultural survey (including recording inputs and outputs from 721 plots) and wild-harvested product survey. The data have been archived with ReShare (UK Data Service). Together these allow a deeper understanding of the household economy on the forest frontier in eastern Madagascar and their swidden agricultural system, the benefits households derive from the forests through wild-harvested products, and the costs of conservation restrictions to forest edge communities.

## Background & Summary

Tropical forests provide vitally important ecosystem services and support valued biodiversity^1–3^. As a result there are many national and global policies seeking to reduce deforestation^4,5^. However, forest conservation can result in real costs to forest frontier communities, many of whom are poor and marginalised, by preventing agricultural expansion and restricting access to valued wild-harvested products^6,7^. International conservation policies recognise that conservation should not make local people worse off, and conservation funded by multilateral donors is also subject to stringent standards to ensure poor people are not made worse off by such investments^8^. However, there is relatively little empirical data allowing a robust evaluation of the costs of conservation for comparison with any benefits distributed.

While forest-dependent people are difficult to define and therefore to count^9^, many millions of people living on the forest frontier in tropical countries make their living from small-scale swidden agriculture and harvesting products from the wild.^10,11^ In many areas these livelihoods face rapid change because of increasing land constraints and reductions in the availability of wild-harvested products due to increases in human populations and over exploitation of previously-abundant species^12–15^ as well as conservation restrictions. In other areas, agricultural intensification and increased salaries for off-farm jobs is resulting in a shift away from forest-dependant livelihoods^16^. Collecting data on the household economy of forest frontier residents in low income countries can be difficult due to poor market integration and highly diversified income sources^17^. However detailed data on the economy of such households is needed to understand the likely impact of potential policies, including protected areas and REDD+ (Reducing Emissions from Deforestation and forest Degradation).

The datasets and surveys described in this paper were part of the project Can Paying 4 Global Ecosystem Services Reduce Poverty? (p4ges) (http://p4ges.org/). The p4ges project ran from 2013 to 2018 and was funded under the Ecosystem Services for Poverty Alleviation (ESPA) programme (www.espa.ac.uk). P4ges aimed to influence the development and implementation of international ecosystem service payment schemes in the interests of poverty alleviation. The project focused on the case study of a new protected area and REDD+ pilot project in eastern Madagascar: the Corridor Ankeniheny Zahamena (CAZ). The World Bank funded the establishment of the CAZ protected area^18^. This meant that households identified as Project Affected Persons (PAPs) were eligible for compensation under the World Bank’s social safeguard scheme for economic displacement caused by the conservation of the area^19^.

This paper presents the datasets created for estimating the magnitude and distribution of net local welfare impacts from the conservation approaches taken in and around CAZ. The data was collected in two phases. In the first phase we conducted household surveys with a stratified random sample of 603 households across five sites. We collected information on demographic and basic socio-economic characteristics of the households, including land holdings, general information on the use of wild-harvested products, whether the household had received compensation under the World Bank social safeguard scheme, assets and wealth indicators, and information on social and human capital. Alongside these interviews we also conducted a choice experiment, designed to estimate the opportunity cost of conservation restrictions which prevent households from clearing further agricultural land from forests.^20,21^ The second phase collected much more detailed information on land use, agricultural practices, off-farm income and wild-harvested products with a stratified random sub-sample of 171 households across four sites.

The datasets described here are already providing rich information for the analysis of opportunity costs of forest use restriction,^20–22^ evaluating the implementation the World Bank social safeguards,^22,23^ and patterns of migration and the role of migration in land use change^24^. They will also allow opportunity cost estimates using the household production function approach, in-depth analysis of forest dependency and the swidden agricultural system practiced in this area, including how crop production varies with the land use history of a plot.

## Methods

**Figure 1:**
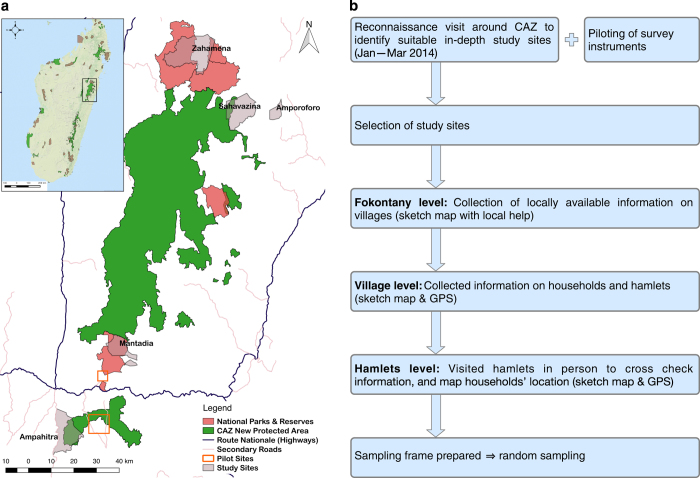
Selection of study sites and sampling. (**a**) Map of the Corridor Ankeniheny-Zahamena (CAZ) new protected area indicating the location of existing protected areas and our study sites. (**b**) Schematic showing the systematic site selection process and development of sampling frame in each site.

This section introduces the case study, the rationale for the selection of study sites, the sampling strategy, the sampling frame, ethical considerations and the implementation of both phases of data collection. Figure 1 provides a map of the study area with pilot and study sites (a), and an overview of the site selection and sampling strategy (b) while Table 1 summarises the study sites selected.

### The case study

The Corridor Ankeniheny Zahamena (CAZ) is a 382,000 ha belt of rainforest linking a number of existing protected areas (most notably Zahamena and Mantadia National Parks) in eastern Madagascar. The CAZ, which is managed by Conservation International on behalf of the Malagasy government, was initially granted temporary protected status in 2006. Its status as an IUCN category VI protected area was confirmed in April 2015. More than 60,000 people live in more than 450 villages in and around this protected area and rely primarily on swidden agriculture, and on collecting wild-harvested products for their livelihoods^18^. As an IUCN category VI protected area, local residents are allowed to collect some forest products for personal use from parts of the protected area. However, swidden agriculture is completely prohibited meaning that the majority of households will be affected by conservation restrictions imposed due to the new protected status.^22,23^ The CAZ is considered a REDD+ pilot project as carbon finance is part of the long-term funding plan for CAZ^25^. Because the establishment of the CAZ was funded by the World Bank, the CAZ environmental and social safeguards plan^18^ follows the World Bank guidelines in identifying and compensating households considered as Project Affected Persons (commonly known as PAPs). This mandates that anyone who will be economically displaced by the project through restriction of access to the natural resources should be compensated. Compensation took the form of micro-development projects such as improved agriculture, small-scale livestock and beekeeping projects^26^. These were distributed to households identified as PAPs in 2014 (soon after the first phase of our field work).

### Selection of study sites

Five study sites, two adjacent to the CAZ new protected area, two next to adjacent long-established protected areas, and one away from the forest frontier (Fig. 1a), were purposively selected for this study following an extensive reconnaissance during January to March 2014. The reconnaissance included collecting site-level information on which to base the selection of the study sites and extensive key informant interviews (with local leaders, elders and school teachers) to ensure we understood the local context and to explore the availability of appropriate sampling frames at each site.

The two sites adjacent to the CAZ new protected area were selected to assess the impacts of the recent conservation restriction on the households. In these sites, although the forest was officially protected under a temporary conservation order (from 2006), there was little enforcement of conservation rules at the time of our data collection. This allowed us to explore livelihoods (and estimate the opportunity cost of conservation restrictions) where the enforcement of conservation restrictions has not yet been fully implemented. To allow us to investigate the effectiveness of compensation provided to households under the World Bank social safeguard process^18,26^ we intentionally selected one site which had been identified to receive compensation (*Ampahitra;* south-west on the map in Fig. 1a), and one site which had not (*Sahavazina;* north-east on the map in Fig. 1a). We selected two study sites next to the existing protected areas (*Zahamena National Park* and *Mantadia National Park* on the map in Fig. 1a) to allow us to explore livelihoods (and estimate the opportunity costs of conservation restrictions) in communities with long history of conservation. All four sites were similar in terms of the households’ reliance on swidden agriculture as primary mode of production, lack of basic services, access to markets, and accessible roads. Only one site, *Ampahitra*, had a secondary road passing through the *fokontany* centre but the road was virtually unpassable during rainy season, and did not have regular scheduled transport. Moreover, the villages selected for study at this site were away from the road ranging from one to four hours walking distance. Finally, the fifth site (*Amporoforo*; east on the map in Fig. 1a) was selected to allow us to explore livelihoods and the land and forest use pattern in a site where forest had been lost (in this case more-or-less complete deforestation by the year 2000, with most of the deforestation occurring during the period 1973-1990)^27^. This final site was important for understanding how livelihoods might change in response to the loss of forest over time. Each site represented one or two *fokontany* (the smallest administrative unit in Madagascar).

### Developing the sampling frame

The sampling unit in this work was the household — defined as one person living alone or a group of people living together, who pool some, or all, of their resources (labour, income and wealth) and who make common provision for food and other essentials for living^28^. Information on the location of rural communities in Madagascar and the number of households in each is not easily available, and where the data exist, they tend to be poor and not up-to-date^23^. In order to build a robust sampling frame for this study, most of our study sites required a thorough census of the households within these sites. As outlined in Fig. 1b, once the study sites were selected, we worked through various levels of local communities starting from the *fokontany*, down to hamlets to conduct a census of the households in each site for our sampling frame. This process involved starting with available maps and drawing sketch maps with local informants (including the *fokontany* president, village chiefs, elders and school teachers) and then visiting villages and hamlets to record the location of households with a handheld GPS. Given the difficulty in accessing many of our sites, and the location of individual households in difficult, hilly terrain, this process took a lot of time (approximately fifty person-days per site).

### Stratified random sampling

Once we established the sampling frame at each site, stratified by village as settlements were grouped within each *fokontany* in small villages, the households for the initial household survey and choice experiment (phase one) were identified through random sampling from each village stratum in proportion to get a set sample size for each site. We did not stratify by wealth status as it was not locally appropriate to discuss wealth status of households in focus groups for us to be able to collect this information. The sample size for each site was based on the minimum number of households required for the choice experiment analysis^29^, as this exceeded the minimum number of households required for other types of data analysis planned. As a rule of thumb, surveying 50 individuals per experimentally designed alternative is acceptable^30^ in choice experiment surveys. This implies that a minimum of 150 respondents were required for our choice experiment design of three alternatives per choice set. This is the lowest limit which provides adequate variation in the variables of interest for which robust models may be fitted. However, other aspects also determined the sample size: the number of choice sets per respondent, the number of attributes and levels, the task complexity, and the field conditions. We administered six choice sets per household in total (with four attributes of varying levels per choice set) and used a minimum sample of around 100 households per site where choice experiment was conducted. We needed a particularly large sample for the choice experiment in *Ampahitra* as we were testing willingness-to-pay and willingness-to-accept formulations^20^ before settling on the best performing approach for roll out across sites. As choice experiments were not being conducted in *Amporoforo* (there is no forest easily accessible from this site meaning it did not make sense to explore the opportunity cost of conservation in this way), the sample size in this site reflects the number of households needed in phase two of the survey. We randomly selected 10–15% more households than needed for our survey in each site to allow for refusals. If a household did not want to be interviewed, we turned to the next household from our sample. Refusals and dropout rates were very low (less than 4% across all sites).

### Ethical considerations and procedures

The surveys covered potentially sensitive subjects such as wealth indicators and land holdings. As clearing forest for swidden agriculture is forbidden in the study area, questions about land use and the history of swidden agricultural plots are particularly sensitive. Given we needed to record individual identifying information (to allow us to relocate households and tie their information together for the in-depth phase two interviews with a sub-sample of those initially interviewed), we had to be particularly careful to ensure that informants’ data was protected. Bangor University's College of Natural Sciences ethics committee approved this study, including the information sheet, consent protocol and the data collection protocol. All members of the survey team received ethics training before carrying out fieldwork, which covered topics such as the principle of voluntary participation, informed consent, and anonymity and data protection. At the beginning of each survey, we introduced the research team and the project objectives to the respondents, why they had been selected for interview, and explained that their participation in the research was voluntary and they could leave at any time. We explained that no information that could identify households or individuals would be shared outside the research teams at Bangor University and the University of Antananarivo and that the research would be used to help others understand about life in their village and how decisions made about conservation might influence them. We gave each household a leaflet to keep which explained the aims of the research in the local language and contained contact details for those responsible for the project with photos and names of the research team overleaf. Details of the consent forms and the information sheet are provided with the archived data. Both archived datasets described in this paper are completely anonymised. We collected some qualitative information alongside the quantitative data recorded in the archive. Given the sensitive nature of the issues discussed and difficulty in anonymising these qualitative information, they have not been archived alongside the quantitative datasets.

Participants in the household survey and choice experiment (phase one) were given a small gift of useful items to a total value of 3,000 ariary (approximately $1) as a gesture of appreciation at the end of the survey. Before giving this gift we asked households if they would be willing to be included in our sample for follow up interviews. The detailed agricultural surveys (phase two) took a full day and required the household head to work as a guide for the day taking us to their fields so we paid the daily wage rate of 5,000 ariary (approximately $1.85). The follow up wild-harvested product survey was compensated in the same way as the initial household survey and choice experiment.

### Survey implementation

**Figure 2:**
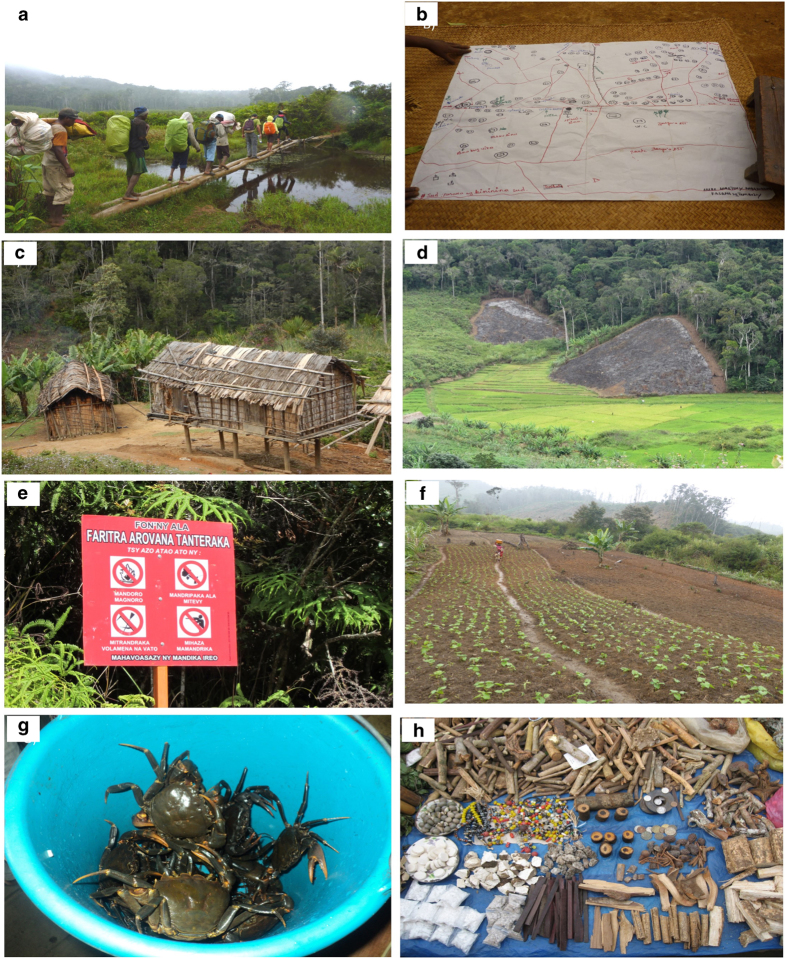
Images showing the field work context. (**a**) Many of the communities visited during this work were remote and only accessible on foot. (**b**) Due to lack of a robust sampling frame we used local knowledge to update available maps and then visited every village and hamlet. (**c**) Most people in the area are poor with most houses made of locally available materials. (**d**) A swidden agricultural system (known locally as ‘tavy’ is practiced throughout the study area. (**e**) Conservation restrictions prevent expansion of agricultural land or collection of most wild-harvested products. (**f**) Micro-development projects such as improved bean cultivation have been offered by the authorities in compensation for conservation costs. (**g**) & (**h**) Wild-harvested products including freshwater crabs and products used for medicine and flavouring are important to local livelihoods.

Figure 2 shows images of the data collection. These are useful for understanding the context.

Phase one (household survey and choice experiment) and the first round of phase two (agricultural surveys) were implemented collaboratively by Bangor University (School of Environment, Natural Resources and Geography) and the University of Antananarivo (Ecole Supérieure des Sciences Agronomiques). The follow up wild-harvested product survey was implemented by Madagasikara Voakajy in close collaboration with the Bangor University and University of Antananarivo teams. Our team spent more than 150 person days in each site, usually with two or three people in the field together. We stayed with members of the community rather than camping separately or travelling in every day. This helped greatly in building relationships and trust.

Extensive field testing of the survey instruments was carried out in order to refine and polish the questions and ensure they could be well understood by our target population and that respondents were comfortable answering them. The pilot sites for testing of the survey instruments were chosen such that these resembled the actual study sites in their characteristics (see Fig. 1a–indicated by orange-coloured squares). Initial survey instruments for all phases were first piloted during February and March 2014 in the pilot study sites. The survey questionnaires were tested both on individual respondents (over 20 in total) and relevance of the questions discussed with focus groups (3-4 in each pilot site). Based on these pilots, survey questionnaires were modified, particularly the sections related to the wealth indicators, land use and types of crops farmed, collection of wild-harvested products and social capital. For the DCE survey, initial piloting helped decide on the key attributes and their levels for the final design of the choice sets. The CE presentation and framing (lengthy warm up steps and use of dolls and large images to desensitise forest clearance) were also significantly informed by the piloting^20^. The final survey instruments were tested again in the pilot sites during June 2014 before the main surveys. The final testing was also important for the training of the research assistants.

The bulk of the fieldwork was carried out by a relatively small team (see acknowledgements) of highly trained research assistants who remained involved in the project from the development of the survey instruments, through data collection, cleaning, archiving and analysis. Three additional short-term assistants helped with the initial household survey, and we had one additional assistant helping with the agricultural survey in one site. Interviews were conducted in person with the household heads (or another adult member of the household where household heads were not available). Interviews were conducted in Malagasy by native speakers comfortable with the dialect of the region.

The surveys were implemented between July 2014 and November 2015 (Table 2). We collected the data on paper forms in both phases and for all sites. While we experimented with data entry onto tablet computers in the field, problems with keeping them charged and the challenges of working on small screens meant we entered the majority of data collected after returning from the field. Data was entered by the research assistants themselves after they returned to the capital after each round of interviews in each site. Survey data and accompanying documents including all survey instruments in English and Malagasy are available on the ReShare repository (Data Citation 1; Data Citation 2).

### Phase one (household survey and choice experiment)

The purpose of phase one interviews was to collect demographic and basic socio-economic characteristics of the households, including migration status, land holdings, wealth indicators, general information on the use of wild-harvested products and to estimate the opportunity cost of conservation from a relatively large sample using discrete choice experiment, a stated preference technique (further details below). We also recorded whether the household had received compensation under the World Bank social safeguard scheme. A secondary purpose was to provide information from which to identify a representative sample for the more in-depth interviews in phase two. Interviews in phase one took between one hour and two hours each.

The socio-demographic and wealth indicators in the household survey were developed from a combination of questions from the Poverty and Environment Network (PEN) household surveys^31^ and World Bank’s Living Standards Measurement Surveys (LSMS). Many of the assets and wealth questions had to be adapted by the team to the rural Malagasy context as assets owned by many households are so limited, the standard LSMS items would not separate our households sufficiently. This was based on expert judgement of our team (among our winder team we had people with many decades of experience of field research in rural Madagascar), as well as from the information gathered during pilot surveys. Project-specific questions were added about land use and access to and use of forests. The key variables covered in the survey are shown in Table 3.

**Figure 3:**
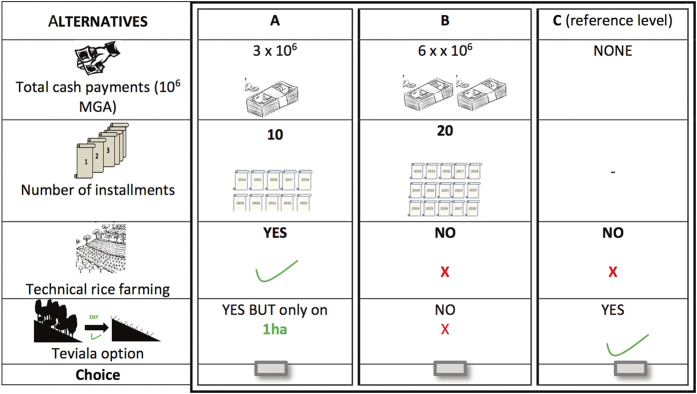
Example of a choice card in the willingness to accept (WTA) format of the choice experiment.

The choice experiment aimed to assess the opportunity costs experienced by households prevented from clearing forest for swidden agriculture due to the introduction of conservation restrictions. The choice experiment used the willingness to accept (WTA) format in all sites, with willingness to pay (WTP) format also being conducted in one site to compare the two formats^20^. Choice experiment is rooted in Lancaster’s model of consumer choice, which proposed that consumers derive satisfaction not from goods themselves but from the attributes they provide^32^. For example, a lake can be decomposed in terms of its turbidity, its recreational facilities (fishing, swimming), and its ecological quality. Changing attribute levels will essentially result in a different good being valued, choice experiments therefore focus on changes in these attributes. The attributes and levels in our surveys were informed by three focus group discussions and pilot testing of the design with 50 respondents. Based on the focus groups and pilot tests, four attributes with varying levels were included in the final choice tasks: i) Total cash donations (framed as development assistance) or total payments made to the government (in WTP format); ii) Number of annual instalments over which the household would receive/pay the cash; iii) Support for improved rice farming; and iv) Clearance of new forestlands for agriculture. The forest clearance attribute had three levels: free clearance (forest protection is lifted), permit for one hectare of clearance, and no clearance (strict enforcement of forest conservation). We combined alternative levels of the four attributes in choice tasks using an efficient design that seeks to minimize the standard error of the coefficients to be estimated^33^, and optimised the fractional factorial design for d-efficiency for the multinomial logit model based on information on the signs of the parameters obtained from the piloting^34^. The design generated 12 choice tasks which were divided into two blocks of six choice tasks each; with each respondent randomly assigned one of the two blocks in the experiment. An example of a choice task is presented in Fig. 3, and more detail of the choice experiment is available in documentation included with Data Citation, 1 and in citations 20, 21 and 29.

At the end of the survey respondents were asked if they would be willing to be interviewed in further surveys. 94% agreed to be included in our sampling frame for phase two.

### Phase two (in-depth agricultural survey and wild-harvested product survey)

Phase two interviews were carried out with a stratified random sample of the phase one households. We stratified households according to household size and land-holding and randomly sampled 40–50 households to represent these strata. The agricultural survey often took a whole day to complete while the wild-harvested product follow-up survey took between 30 minutes and 1.5 hours.

#### Agricultural survey

The agricultural survey comprised of two major sections – (i) general household-level information, including land ownership, land access, land use, livestock inputs and outputs, off-farm income, and information on clearance of primary forest; and (ii) field (plot) level data on the history of each individual plot (when it was cleared from forest, crop cycles) and inputs and outputs over the last complete agricultural year. We visited all accessible plots and mapped the boundary of over 80% of those with a handheld GPS in order to understand the distribution of land holdings relative to the forest frontier and to calculate the size of each plot (649 visited, 520 mapped; out of 898 plots recorded). Field level data for all the accessible plots was recorded at the plot location to aid recall, and in-depth agricultural inputs-outputs data was collected for all the plots cultivated by the households during the previous full farming year (721 in total). Overall, we collected plot level data for 898 plots belonging to the 171 households.

Cognisant of the potential limitations of the recall surveys, we identified a number of households in the site Zahamena who agreed to keep agricultural logs (daily or weekly) for the ongoing agricultural year to allow us to investigate the reliability of the recall in the agricultural surveys. These logs covered the same information as in the agricultural inputs-outputs and off-farm income surveys, however, recorded either daily or weekly. The formats used by the households to keep these logs and the data produced are archived alongside the main data. Where inputs and outputs were recorded in local units, these have also been converted to standard units in the dataset. Information on specific conversion factors were gathered at the community level from local markets and through measurement by researchers in the field as required.

Finally, we carried out yield measurement for hill rice for a selected number of plots during the harvest period in Zahamena. The selection of these plots was opportunistic rather than random based on the plots ready for harvesting during our team’s visit to the site at harvest period. A total of 11 hill rice plots were selected for yield measurement. To estimate the plot yield, we randomly selected ten (10) 1m^2^ quadrats in each plot, harvested the rice from these quadrats with the help of members of the households farming the plot, and measured the harvest both in standard and local units. The average yield from the 10 sample quadrats were used to extrapolate for the whole plot using the plot area.

#### Wild-harvested product survey

We attempted to reach all households who were interviewed in the agricultural survey in the wild-harvested product survey (carried out a few months later in each site). We managed to reach all but two households meaning that the sample size in these follow up surveys covered 169 of the 171 households targeted.

Before conducting the wild-harvested products survey in each site we carried out focus group discussions to identify a prioritised list of the most important wild-harvested products in each site. The survey then gathered data on each of the wild-harvested products on the prioritised list plus additional products that a household considered important. Data collected included seasonal collection of wild-harvested products, their use and sale, their importance to the livelihood of the household, and abundance historically and perception regarding their likely availability in the future.

### Code availability

This study did not use any computer codes to generate the dataset. Microsoft Excel was used to enter, store and quality check the collected data. Where new variables and data associated are calculated/derived from other survey variables, these have been detailed in metadata with the functions used to derive these variables.

## Data Records

Two archived datasets, both containing primary data and accompanying documentation, relate to this data paper (see Table 2). The first covers phase one (the household survey and choice experiment) (P4GES_HHS_CE.zip, Data Citation 1). The data was collected between June 2014 and June 2015 and comprises of 603 households. The second covers phase two (the agricultural survey and wild-harvested product survey) (P4GES_AGR_WHP.zip, Data Citation 2).

## Technical Validation

A number of steps were employed from the beginning of the study to ensure quality of the data collected and that of the datasets deposited in the archive. First we conducted extensive reconnaissance including key informant interviews to ensure our research team (many of whom had experience of the study region prior to this project) had a good understanding of the local context to inform development of the survey instruments. We also conducted an extensive pre-testing of questions to ensure they were relevant and well understood locally.

Secondly, our research team included native Malagasy speakers and only one of the non-Malagasy involved in designing the survey and developing protocols in the field does not speak Malagasy. The Malagasy version of the survey instruments were therefore developed alongside the English version and we constantly discussed how particular concepts would be communicated in Malagasy. The lead researcher from Bangor University who developed the choice experiment (OSR) is a native Malagasy speaker which was vitally important in ensuring that this challenging survey was developed in a way which could be effectively understood by respondents. The validity of the choice experiment was explicitly tested before being rolled out across study sites.^20,21^ The archived English version of the instruments were reverse translated from the Malagasy version used in the field.

Third, we made a conscious decision not to depend on a large team of enumerators, hired after the surveys were developed, trained and then deployed to collect data. Instead we worked closely with a relatively small group of research assistants, the majority of whom already had experience in the study area and experience of social research and the majority of which stayed with the project for at least three years (through reconnaissance, development of the survey instruments, piloting, data collection, cleaning and archiving). Many of these research assistants are co-authors on the papers coming out of this data set. In phase one surveys; this core team were helped by between one and three assistants (depending on the site). We provided comprehensive training to everyone involved in data collection. The acknowledgements section provides the names of everyone involved in data collection.

Fourth, the senior researchers (the authors of this paper) closely supervised data collection in the field, particularly at the beginning of the work in each phase at each site. The lead researcher who developed the choice experiment (OSR) was in the field for the whole of phase one (household survey and choice experiment) at two of the sites (*Ampahitra* and *Mantadia;* three months in total). MP was also regularly in the field for quite extended periods of time; spending eight weeks in the field in total while JPGJ, NH and JHR made shorter visits.

Fifth, at the end of each survey our team recorded answers to questions relating to their perception of how well the interview had gone including how open and engaged the respondent seemed and how well they had understood the process. They also kept daily logs based on their interviews and interactions with the respondents, and other general observations in the field, and compared notes with each other on a daily basis. These were helpful in learning from each other, and in resolving any minor issues during the surveys.

Finally, a great deal of time and effort was put into data entry, data checks and validation. For majority of the cases, the research assistants who carried out the surveys entered the data for the households that they had interviewed. This raw data was then checked systematically by another member of the team for any errors or inconsistencies. The data was then coded for clarity and consistency. The coded data was checked further by senior members of the research team - checking specially for potential typing errors and extreme values. Where inconsistencies and potential errors were located, these were checked against the original raw data as well as the original paper survey sheets to get to a resolution.

## Usage Notes

### Data access conditions

Individual household-level data has been anonymised before submitting to the archives and we have not included GPS locations of the household (converting this data to ‘distance to the forest frontier’). However, given the detailed nature of the data collected at the household level, particularly regarding their household demography, and on- and off-farm income activities, there exists certain level of sensitivity surrounding the data. This potentially creates a situation where households within each survey sites, particularly those with small sampling frames, can be identified from the survey data. For this reason, both datasets described in this paper have been made available as safeguarded data on the UK Data Archive’s data repository ReShare. Anyone wishing to download and use these data must register with the UKDA and agree to the conditions of their End User Licence, outlined at https://www.ukdataservice.ac.uk/get-data/how-to-access/conditions#/tab-end-user-licence. For commercial use, please contact the UK Data Service at help@ukdataservice.ac.uk.

### Variables included in both phase one and two

Some questions relating to land use and land holdings and the collection and use of wild-harvested products were asked to the respondents in both phase one and phase two so for the subsample interviewed in both phases, some of this information will be repeated in the two datasets. For a general analysis using a large sample size, phase one data is most appropriate, but phase two data allows for more in-depth analysis.

## Additional information

**How to cite this article:** Poudyal. M. *et al.*, Household economy, forest dependency & opportunity costs of conservation in eastern rainforests of Madagascar. *Sci. Data*. 5:180225 doi: 10.1038/sdata.2018.225 (2018).

**Publisher’s note:** Springer Nature remains neutral with regard to jurisdictional claims in published maps and institutional affiliations.

## Supplementary Material



## Figures and Tables

**Table 1:** Characteristics of the study sites and rational for their selection.

Sites	Fokontany(s) (Commune) DISTRICT	Protected status	History of conservation	Enforcement of conservation rules∗	Compensation provided
Mantadia	Volove & Vohibazaha (Ambatavola) MORAMANGA	Established Protected Area	Long history of conservation (since 1989) on periphery of Mantadia National Park	Relatively strong	Park entry fees shared with community and used to fund local development projects
Zahamena	Antevibe & Ambodivoangy (Ambodimangavalo) VAVATENINA	Established Protected Area	Long history of conservation (since 1927) on periphery of Zahamena National Park	Relatively strong	Park entry fees shared with community and used to fund local development projects
Ampahitra	Ampahitra (Ambohibary) MORAMANGA	New Protected Area (limited experience of conservation)	Granted temporary protected status in 2006, formally gazetted in 2015.	Weak	Yes (World Bank social safeguards for new protected area)
Sahavazina	Sahavazina (Antenina) TOAMASINA II)	New Protected Area (limited experience of conservation)	Granted temporary protected status in 2006, formally gazetted in 2015.	Very weak	No
Amporoforo	Amporoforo (Amporoforo) (TOAMASINA II)	Not applicable (not on forest frontier).	The forest at this site was lost in the 1950s and there is no conservation effort.	Not applicable	Not applicable

^∗^Information based on field observation during site reconnaissance and our knowledge based on interactions with conservation organisations over the past five years.

**Table 2:** Timing, recall period, sampling frame, and sample size (number of households surveyed) in each survey phase.

Phase one: household survey and choice experiment (Data Citation 1; N=603)				
Site	Period of survey	Recall period∗	Sampling frame/sample size†	Households surveyed
Ampahitra	Jul‒Aug 2014	Sep 2013‒Jun 2014	431/260	203
Mantadia	Aug‒Sep 2014	Sep 2013‒ Jun 2014	241/141	104
Zahamena	Oct‒Nov 2014	Sep 2013‒Jun 2014	673/175	152
Sahavazina	Feb 2015	Sep 2013‒Jun 2014	409/125	95
Amporoforo‡	Feb‒Mar 2015	Sep 2013‒Jun 2014	175/50	49
**Phase 2a: Agricultural survey (** Data citation 2**; n=171)**				
**Site**	**Period of survey**	**Recall period**∗	**Households surveyed**	
Ampahitra	Aug‒Nov 2014	Sep 2013‒Jun 2014	50	
Zahamena	Nov 2014‒May 2015	Sep 2014‒May 2015	41	
Sahavazina	May‒Jun 2015	Sep 2014‒May 2015	40	
Amporoforo	Jun 2015	Sep 2014‒May 2015	40	
**Phase 2b: Wild-harvested product survey (**Data citation 2**; N=169)**				
**Site**	**Period of survey**	**Recall period**	**Households surveyed**	
Ampahitra	Oct‒Nov 2014	Last 12 months	50	
Zahamena	Aug‒Sep 2015	Last 12 months	40	
Sahavazina	Oct 2015	Last 12 months	40	
Amporoforo	Nov 2015	Last 12 months	39	

^∗^Recall period refers to the period of inputs and outputs relating to agricultural and off-farm livelihood activities. For householdsʼ demographic and other socio-economic data, such as wealth indicators data collected related to the situation at the time of survey.

^†^This includes number of households randomly sampled including 10–15% replacement allowance.

^‡^In Amporoforo, choice experiments were not planned so we limited the sample size to what was required for phase 2 of the surveys in this site.

**Table 3:** The main sections of the household survey showing the groups of variables collected.

Section	Information included
Information about the household	Place of birth and ethnicity of HH head, when household was formed, marital status of household head, detailed roster of all members of the household including age, sex, relation to the household head, education and occupation
Land ownership, land access and land use	Households are asked to list all the plots they had access to in the last agricultural year and for each to provide information on: the type of field, the tenure situation, plot age, fertility and size. For plots which are loaned or rented there are additional questions about who they are loaned or rented to. They were also asked whether they could have cultivated more land with the resources available and if not, why they didn’t cultivate more land.
Wild-product harvest	For each of the categories (firewood, roofing material, material for walls, timber, material for floor and weaving materials) we asked them to report on their preferred species, the type of land they collect them from, and whether they were used in the household or sold. They were also invited to provide such information for other categories of wild-harvested products important to them.
Compensation provided for the costs of conservation	Households were asked whether they took part in the safeguard assessment in 2009/2010, whether they were identified as a project affected person, whether they received a micro-development project under the social safeguard scheme, and what the project was.
Assets and wealth indicators	We collected information on the number of rooms, roof and floor construction of their main house and any ‘field houses’, the number of each category of livestock owned, and whether they owned each of a list of household items. We also asked the number of months they had enough to eat in the last farming year, the type of light used in the house and how often they had sufficient light.
Social and human capital	They were asked about training courses attended by any member of the household and membership of community groups. They were also asked a series of questions aimed to evaluate support networks e.g. If I have serious problems (losing crops/illness etc.), I get help from my neighbour/wider community.

## References

[d1] Colchester, Essex: UK Data Archive Poudyal M. *et al.* 2018 https://dx.doi.org/10.5255/UKDA-SN-852435

[d2] Colchester, Essex: UK Data Archive Poudyal M. *et al.* 2018 https://dx.doi.org/10.5255/UKDA-SN-852790

